# Diversity of microbial carbohydrate-active enzymes in Danish anaerobic digesters fed with wastewater treatment sludge

**DOI:** 10.1186/s13068-017-0840-y

**Published:** 2017-06-21

**Authors:** Casper Wilkens, Peter Kamp Busk, Bo Pilgaard, Wen-Jing Zhang, Kåre L. Nielsen, Per Halkjær Nielsen, Lene Lange

**Affiliations:** 1 Center for Bioprocess Engineering, Department of Chemical and Biochemical Engineering, Technical University of Denmark, Søltofts Plads, Building 229, 2800 Kongens Lyngby, Denmark; 2 Section for Sustainable Biotechnology, Department of Chemistry and Bioscience, Aalborg University, A. C. Meyers Vænge 15, 2450 Copenhagen, Denmark; 3 Center for Microbial Communities, Section for Biotechnology, Department of Chemistry and Bioscience, Aalborg University, Frederiks Bajer Vej 7, 9220 Aalborg, Denmark; 4 Department of Animal Sciences, University of Wisconsin-Madison, Madison, WI 53706 USA

**Keywords:** Carbohydrate-active enzymes, Anaerobic digester, Wastewater treatment, Bacteria, Glycoside hydrolase, Glycosyltransferase, Carbohydrate esterase, Polysaccharide lyase, Carbohydrate-binding module, Metagenomics, Enzyme discovery, Peptide pattern recognition, Carbohydrate hydrolysis

## Abstract

**Background:**

Improved carbohydrate-active enzymes (CAZymes) are needed to fulfill the goal of producing food, feed, fuel, chemicals, and materials from biomass. Little is known about how the diverse microbial communities in anaerobic digesters (ADs) metabolize carbohydrates or which CAZymes that are present, making the ADs a unique niche to look for CAZymes that can potentiate the enzyme blends currently used in industry.

**Results:**

Enzymatic assays showed that functional CAZymes were secreted into the AD environments in four full-scale mesophilic Danish ADs fed with primary and surplus sludge from municipal wastewater treatment plants. Metagenomes from the ADs were mined for CAZymes with Homology to Peptide Patterns (HotPep). 19,335 CAZymes were identified of which 30% showed 50% or lower identity to known proteins demonstrating that ADs make up a promising pool for discovery of novel CAZymes. A function was assigned to 54% of all CAZymes identified by HotPep. Many different *α*-glucan-acting CAZymes were identified in the four metagenomes, and the most abundant family was glycoside hydrolase family 13, which contains *α*-glucan-acting CAZymes. Cellulytic and xylanolytic CAZymes were also abundant in the four metagenomes. The cellulytic enzymes were limited almost to endoglucanases and *β*-glucosidases, which reflect the large amount of partly degraded cellulose in the sludge. No dockerin domains were identified suggesting that the cellulytic enzymes in the ADs studied operate independently. Of xylanolytic CAZymes, especially xylanases and *β*-xylosidase, but also a battery of accessory enzymes, were present in the four ADs.

**Conclusions:**

Our findings suggest that the ADs are a good place to look for novel plant biomass degrading and modifying enzymes that can potentiate biological processes and provide basis for production of a range of added-value products from biorefineries.

**Electronic supplementary material:**

The online version of this article (doi:10.1186/s13068-017-0840-y) contains supplementary material, which is available to authorized users.

## Background

The use of biomass to produce more food and feed from biomass and renewable biobased chemicals, materials, and fuel requires improved carbohydrate-active enzymes (CAZymes) and/or still unknown CAZyme functions that can be added to the current enzyme cocktails used in the industry in order to optimize valorization of the biomass [1]. CAZymes can be identified by studying how microbial communities metabolize the biomass [2]. Unfortunately, only a fraction of the total microbial diversity can currently be cultured in the laboratory [3]; however, metagenomics, combined with use of synthetic genes for production of CAZymes through heterologous expression, has revolutionized our opportunities for CAZyme discovery from microbes often living in complex communities. This enables us to study microbial communities [2, 4–6] and also finds new enzymes for industrial uses.

Metagenomics has been used both to discover novel CAZymes and expand our understanding of the synergism between these enzymes in microbial biomass degradation. A study of switchgrass degraded in cow rumen showed that only 12% of the identified CAZymes had 75% or more identity to known proteins and 43% had less than 50% identity to known proteins. The large fraction of new enzymes demonstrates that metagenomic studies serve as an excellent starting point to discover novel CAZymes [7]. Other metagenomics studies of microbial habitats, which differs in, e.g., temperature, pH, and oxygen availability also showed great variation in CAZymes [8–12]. Despite the plethora of sequencing data already analyzed, new activities within CAZyme families are continuously discovered and even new families established [13–16]. There is still much to uncover about the variety of CAZymes and their accessory proteins [1, 5]. Exploring habitats that differ significantly from already explored habitats should therefore not be neglected in pursuit of novel and more efficient CAZymes. The microorganisms in unexplored habitats may have evolved novel CAZymes in order to cope with both deconstruction of the biomass they use as carbon source and the environment their secreted enzymes must act in.

Such a unique niche is found in anaerobic digesters (ADs) fed with primary and surplus sludge from municipal wastewater treatment plants that is largely unexplored in terms of diversity of CAZymes [17–20]. ADs at municipal wastewater treatment facilities are getting more common due to a shift from only wastewater treatment to also including renewable energy production in the form of methane in the biogas produced. The ADs are complex ecosystems and the hydrolytic bacteria are extremely diverse in ADs, which give the advantage of enormous metabolic flexibility [21]. However, little is still known about the carbohydrate metabolism in ADs and the CAZymes that are present as most metagenomic studies have focused on microorganism composition [22].

Here we explore four mesophilic ADs fed with surplus and surplus sludge from wastewater treatment plants for CAZymes by mining the metagenomes obtained by Illumina sequencing with Homology to Peptide Patterns (HotPep). Peptide Pattern Recognition (PPR) is a non-alignment-based approach that identifies a set of short conserved sequences, which can be used as a fingerprint when mining genomes with HotPep for CAZymes and also to predict the function of the individual enzymes [23].

## Methods

### Sample collection and DNA extraction

Biomass samples were collected from 4 full-scale Danish mesophilic AD reactors at the wastewater treatment plants Søholt, Randers, Viborg, and Frederica. All reactors have been in operation for several years and the first three receive primary sludge (precipitated wastewater) and surplus activated sludge, approx. 50% of each. In Fredericia, the feed is only surplus activated sludge and it is pretreated by thermal hydrolysis. Further details about the plant design and operation can be found elsewhere [24]. The samples were frozen and stored until analysis. DNA was extracted from the biomass using the FastDNA Spin kit for soil (MP Biomedicals, Santa Ana, CA, USA), following the standard protocol except for four times increased bead beating duration and a sludge input volume of 50 μl [25].

### Illumina sequencing and assembly

Illumina TruSeq DNA PCR-free libraries were prepared from DNA extracts according to the manufacturer’s protocol and paired-end sequenced on the Illumina HiSeq 2000 platform (2 × 150 bp) and Illumina MiSeq platform (v3 chemistry, 2 × 300 bp). Reads were quality-trimmed and filtered using default settings in CLC Genomics Workbench (v. 7.5.1; CLC Bio, Aarhus, Denmark). The metagenomic reads were assembled separately for each plant using default settings in CLC Genomics Workbench.

### Mining and annotation

HotPep [26, 27] were used to identify CAZymes in the four metagenomes using peptide patterns generated with PPR in January 2015 for all families of glycoside hydrolases (GHs), auxiliary activities (AAs), polysaccharide lyases (PLs) and glycosyltransferases (GTs) in the CAZy database (http://www.cazy.org) [16]. The identified CAZymes were analyzed for carbohydrate-binding domains (CBMs) and dockerin domains using dbCAN [28]. A BlastP was performed for all identified CAZymes using the NCBI nr database (http://blast.ncbi.nlm.nih.gov/Blast.cgi) to identify the closest related known protein, and BlastP was also used for comparing the CAZymes identified in the four ADs. PhyloPhytiaS was used for taxonomical assignment of the genes coding for the identified CAZymes by searching against “Generic 2013–800 Genera” [29]. The heatmaps were visualized with Multiexperiment Viewer software [30] and Circos software [31].

### Sequence data availability

The four metagenomes were deposited in the National Center for Biotechnology Information (NCBI) and can be accessed in the Whole Genome Shotgun (WGS) under accession numbers MTKY00000000 (Søholt), MTKX00000000 (Randers), MTKZ00000000 (Viborg), and MTKW00000000 (Fredericia). See Table 1 for summary of the sequencing data.

**Table 1:** Sequencing data

	Søholt	Viborg	Randers	Fredericia
Assembly size (Mbp)	255	444	179	283
Nr. of contigs	100,352	141,509	52,793	90,304
N50 (Kbp)	3012	4383	5087	4326

Summery of Illumina sequencing data for anaerobic digesters Søholt, Fredericia, Randers, and Viborg

### AZCL-assay

The enzyme profile of the supernatant for the AD Søholt was investigated using 0.1% insoluble chromogenic AZurine Cross-Linked (AZCL) barley *β*-glucan, pachyman and curdlan, birchwood xylan, wheat arabinoxylan, and galactomannan (all Megazyme) dispersed in 1% agarose plates prepared using the buffer 0.08 M phosphoric acid, 0.08 M acetic acid, 0.08 M boric acid pH 6. The samples were sonicated and the supernatants (15 µl, undiluted) were added to triplicate wells in Petri dishes containing the different AZCL substrates and incubated at 37 °C for 3 days. Enzyme activity was confirmed by a blue halo around the sample well, indicating the presence of active enzymes that can break down the specific AZCL substrate, thereby releasing the blue, soluble dye.

## Results and discussion

### The anaerobic digesters

The four ADs were all typical biogas plants at municipal wastewater treatment plants. Three of them received a mixture of primary sludge (pre-settled wastewater) and surplus activated sludge (Søholt, Randers, Viborg) as feed, while Fredericia received surplus activated sludge only and it was treated by thermal hydrolysis before added to the digester. The reactors have reported loading rates of 1–2.5 kg volatile solids m^−3^ day^−1^, ammonium levels of 500–2400 mg l^−1^, volatile fatty acids (VFA) concentrations of 0.5–15 mmol l^−1^, pH of 7.1–7.8, and sludge retention times of 15–35 days. A recent survey of the microbial community composition in Danish ADs showed that the first three reactors had a very similar composition and it was typical for Danish digesters [32]. Fredericia had a slightly different community composition (unpublished results). Primary sludge is the particulate fraction of wastewater, consisting of approx. 30% lipids, carbohydrates, and proteins [33]. Surplus sludge is the same as activated sludge and it consists primarily of microorganisms [24].

### Secretion of CAZymes in the anaerobic digesters

**Fig. 1:**
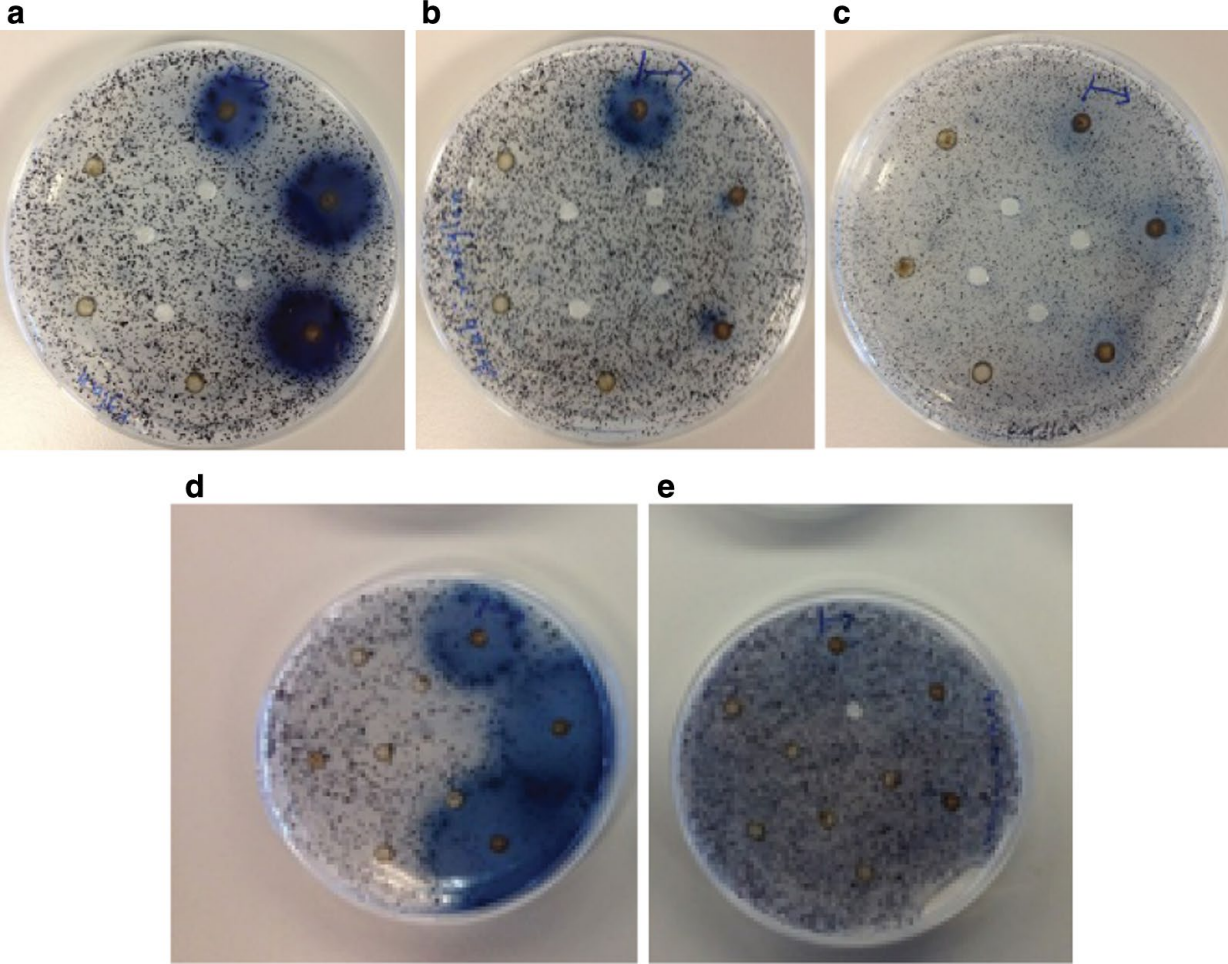
AZCL plates: CAZyme activity screening in AD Søholt **a** birchwood xylan, **b** wheat arabinoxylan, **c** curdlan and pachyman, **d** barley β-glucan, and **e** galactomannan

As preliminary feasibility study, the supernatant of the AD Søholt sampling was tested for CAZyme activities. Activity against birchwood and wheat arabinoxylan was detected (Fig. 1) showing that xylanolytic enzymes were secreted by the microorganisms. Activity against barley *β*-glucan, pachyman, and curdlan (data not shown) suggests the presences of *β*-glucanolytic CAZymes and possibly also cellulytic CAZymes in the supernatant. No activity was detected against galactomannan (Fig. 1). The results indicate that functional CAZymes were secreted into the AD environments.

### Identified carbohydrate-active enzymes

**Fig. 2:**
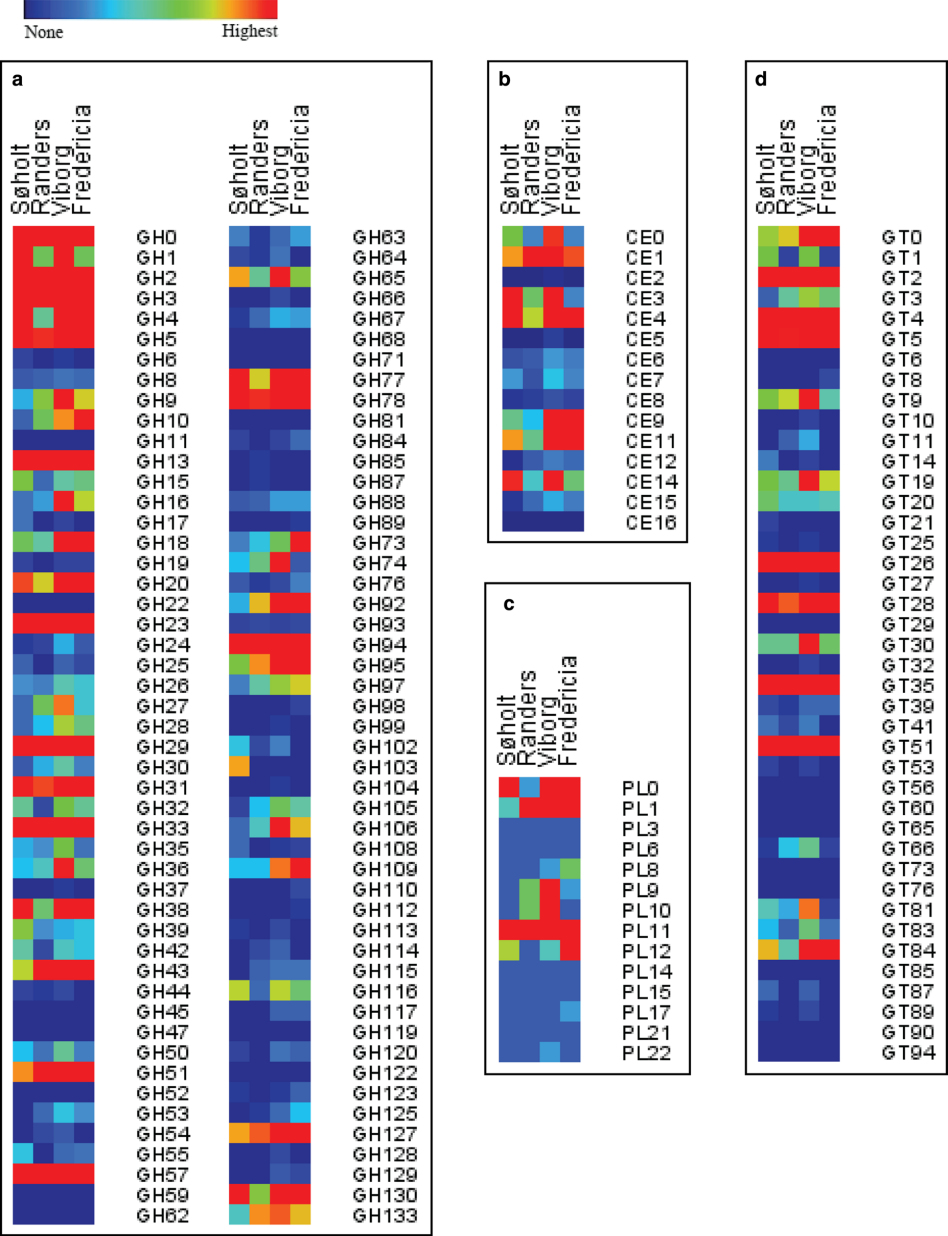
Identified CAZymes: Heat maps of all identified **a** glycoside hydrolases, **b** carbohydrate esterases, **c** polysaccharide lyases, and **d** glycosyl transferases in the metagenomes from the anaerobic digesters (ADs) Søholt, Randers, Viborg, and Fredericia. The enzyme families are listed on the *right side* and named according to the Carbohydrate-Active Database (http://www.cazy.org). The heat maps are calculated individually for each AD and enzyme families with no identified members in any of the metagenomes are not shown

**Fig. 3:**
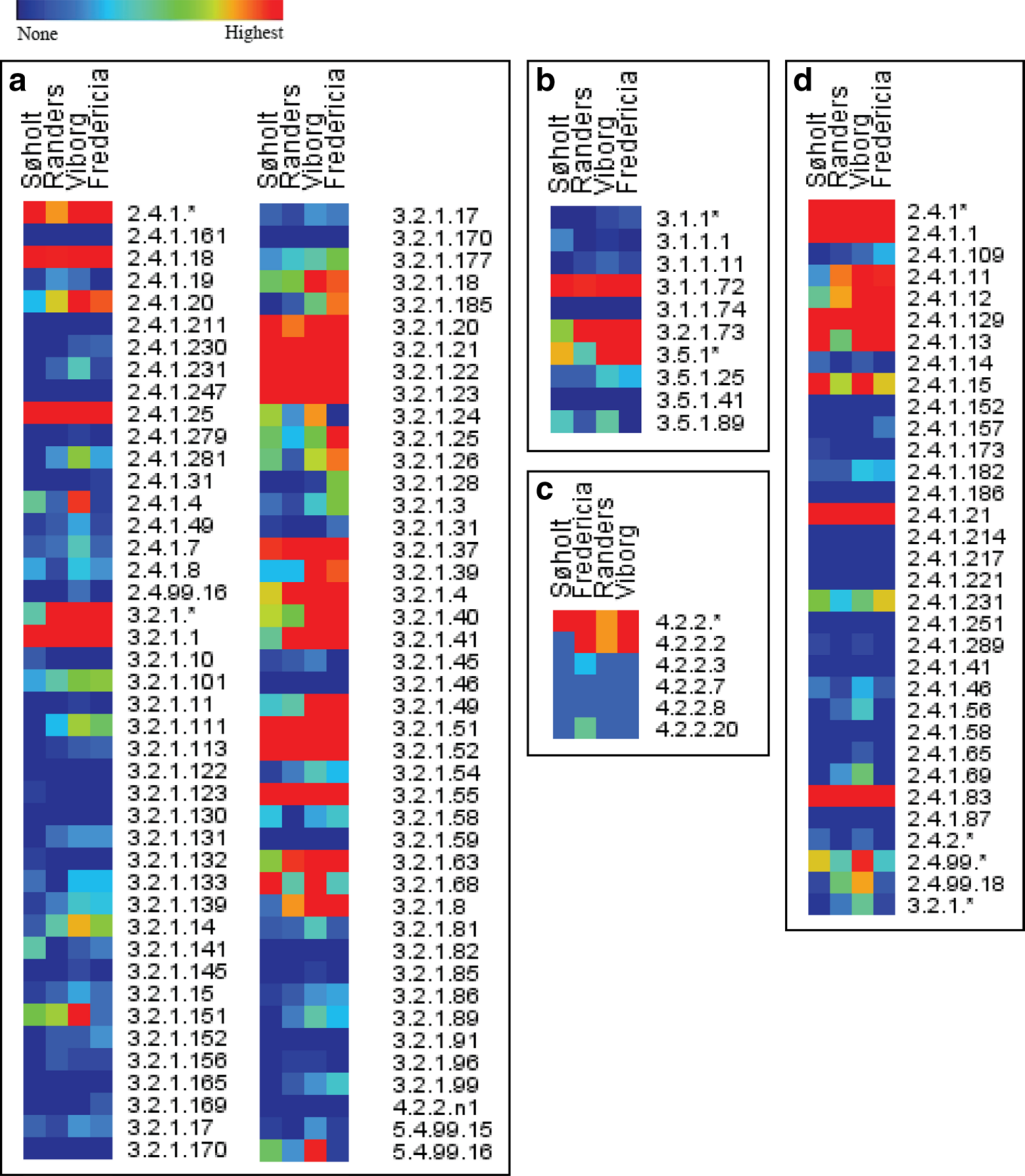
CAZymes with an assigned function: heat maps of all identified **a** glycoside hydrolases, **b** carbohydrate esterases, **c** polysaccharide lyases, and **d** glycosyl transferases in the metagenomes from the anaerobic digesters (ADs) Søholt, Randers, Viborg, and Fredericia to which Homology To Peptide (HotPep) assigned a putative function. On the *right side* are listed the Enzyme Commission (EC) number for the particular type of CAZyme family. The heat maps are calculated individually for each AD

To explore the obtained metagenomes for CAZymes, HotPep [26, 27] was used to mine the four metagenomes for CAZymes. In total 19,335 catalytic domains were identified of which 10,374 were GHs, 6336 were GTs, 2064 were CEs, and 202 were PLs (Table 2). The numbers for the individual ADs are listed in Table 2. 98 of 133 GH, 15 of 17 CE, 14 of 25 PL, and 41 of 99 GT families were represented in the metagenomes (Fig. 2a–d). The diverse repertoire of CAZyme genes (Fig. 3a–d) provides the basis for a flexible carbohydrate metabolism within the microbial community and for discovery of novel CAZymes. A small number (718) of auxiliary activity CAZymes were also identified (Additional file 1); however, these oxidative enzymes have very limited access to oxygen in the ADs. Hence, they are not relevant for the degradation in the anaerobic digesters.

**Table 2:** Nr. of identified CAZymes

	Glycoside hydrolases	Carbohydrate esterases	Polysaccharide lyases	Glycosyl transferases
Søholt	2062 (1297)	457 (178)	44 (13)	1506 (753)
Viborg	3777 (2311)	760 (299)	78 (28)	2451 (1172)
Randers	1573 (1015)	310 (139)	27 (12)	948 (341)
Fredericia	2962 (1996)	537 (209)	53 (20)	1431 (516)
Total	10,374 (6619)	2064 (825)	202 (73)	6336 (2782)

Number of identified glycoside hydrolases, carbohydrate esterases, polysaccharide lyases, and glycosyl transferases in the metagenomes from the anaerobic digesters Søholt, Fredericia, Randers, and Viborg. In the parenthesis is the number of genes for which Peptide Recognition Pattern have assigned a putative function

The CAZymes identified in AD Randers showed low homology to the CAZymes identified in AD Fredericia and AD Søholt; however, high homology to the ones identified in AD Viborg (Table 3; Additional file 2). The CAZymes identified in AD Viborg showed modest homology to the CAZymes identified in AD Søholt and low homology to the CAZymes identified in AD Fredericia, which was also the case when comparing ADs Søholt and Fredericia (Table 3; Additional file 2).

**Table 3:** Percentage of CAZymes with an identity above 90% when compared to the CAZymes from the other ADs

	Randers (%)	Fredericia (%)	Søholt (%)	Viborg (%)
Randers		4	11	75
Fredericia	4		6	3
Søholt	11	6		17
Viborg	75	3	17	

The diversity of CAZyme families has been explored in several anaerobic habitats such as cow rumen [7, 34, 35], muskoxen rumen [36], yak rumen [37], termite gut [38–41], wallaby gut [42], giant panda gut [43], elephant faces [12], and fresh fecal samples from Yunnan snub-nosed monkey [44]. The microbial communities in the four ADs investigated had a higher diversity in identified CAZyme families than found in the above-mentioned rumen and gut samples. This suggests that the microbial community in the ADs (including the microbes coming from the feed to the four ADs) encounters a much more diverse carbohydrate composition than those in the above-mentioned habitats. This shows that the ADs studied is a great place to look for novel microbial enzymes to potentiate the enzyme cocktails used to day for biomass degradation.

Unfortunately, in most of the above-mentioned studies, a specific function is not assigned to the identified CAZymes, and since many CAZyme families include enzymes with different functions, a comparison at family level does not make sense in terms of what the microorganisms found in the ecological niche can degrade. HotPep is trained to assign a function to CAZymes if an activity for a group created by PPR has been determined according to the CAZy database [16]. HotPep assigned a function to 54% of the 19,335 identified CAZymes in the four ADs (Table 2), showing that our knowledge of a lot of the CAZy subfamilies lack behind as HotPep only assigns a function to a PPR group if a member of the PPR group has been characterized [26, 27]. It is reasonable to assume that new specificities could be identified by characterizing CAZymes from these PPR groups.

The four ADs were very similar in terms of percentage of CAZymes with an assigned function (Søholt 56%, Randers 52%, Viborg 54%, and Frederica 55%) (Table 2), suggesting that ADs at municipal wastewater treatment plants in general is a highly promising place to look for novel CAZymes. Looking at the different types of CAZymes, the picture differs as a function has been assigned to 64% GHs, 48% GTs, 40% CEs, 36% PLs, and 25% AAs (Table 2), which reflects the number of characterized members of the different CAZymes.

Low homology to known proteins suggests that the CAZymes could be optimized for specific functions, which could be exploited in industrial process [2]. 30% of the identified CAZymes in the four metagenomes showed 50% or lower identity to known proteins and less than 3% showed 95% or higher identity to known proteins (Additional file 1). This suggests that the ADs at municipal wastewater treatment plants provide a unique and unexploited ecological niche for discovery of CAZymes with novel biochemical and physicochemical properties.

### Microbial origin of the identified carbohydrate-active enzymes

A meta study showed that Chloroflexi, Proteobacteria, Firmicutes, and Bacteroidetes are the dominating phyla in ADs [45]. However, large variations are found within the individual ADs due to differences in the living conditions for the microbes, reactor type, and the biomass the AD are fed with [18, 46–55]. A recent survey of Danish ADs showed that besides the above-mentioned phyla, the 25 most abundant bacterial species are of Actinobacteria and Spirochaetae [32].

**Fig. 4:**
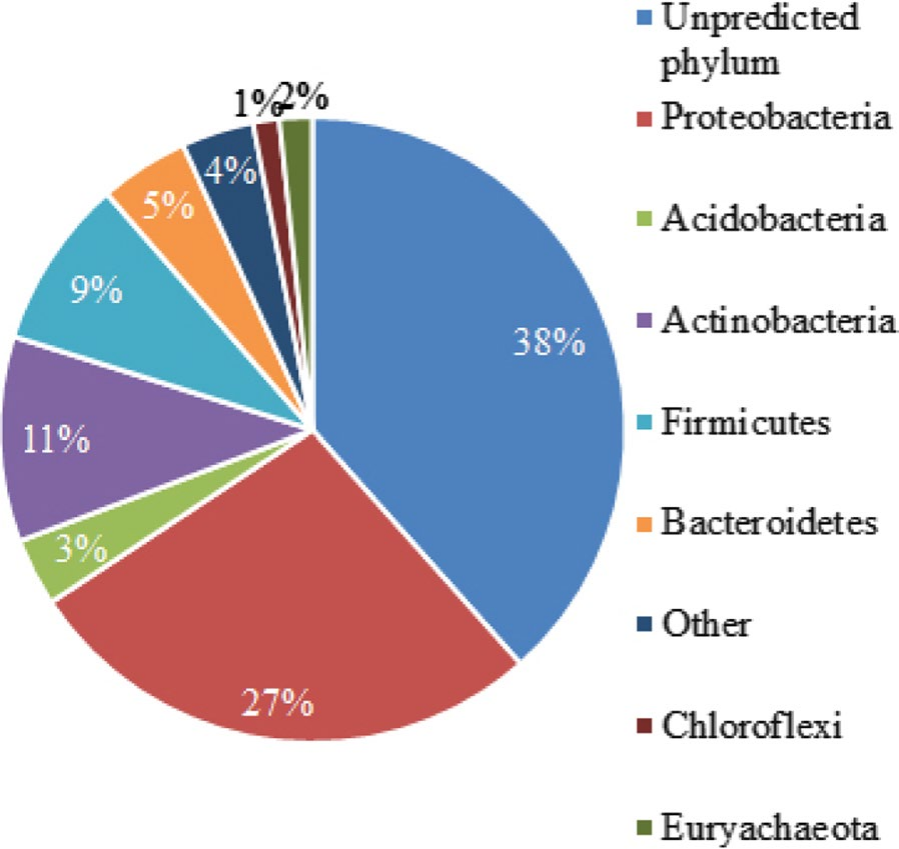
Microbial origin of the identified CAZymes: taxonomic microbial phyla assignment of all identified CAZymes found in the metagenomes from the anaerobic digesters Søholt, Fredericia, Randers, and Viborg

We used PhylopythiaS [29] to investigate which phyla harbored the identified CAZymes from the four ADs. A major part, 38%, of the CAZymes were not assigned to a phylum due to the lack of reference genomes (Fig. 4). Proteobacteria, Firmicutes, Acidobacteria, Actinobacteria, and Bacteroidetes accounted for 55% of the identified CAZymes, while up to 21 other phyla accounted for the remaining CAZymes (7%) (Fig. 4; Additional file 3).

The diverse repertoire and distinct distribution of CAZymes on the bacterial taxonomic groupings within the four ADs suggest that the microbial community within the four ADs is likely to experience changes in the carbohydrate content, compared to, e.g., the uniform diet of ruminants. Hence, this makes the AD a unique place to look for a diverse type of CAZymes.

### Abundance, variety, and taxonomic distribution of *α*-glucan, cellulose, and xylan hydrolyzing CAZymes

We looked into the five dominating phyla; Proteobacteria, Firmicutes, Bacteroidetes, Acidobacteria, and Actinobacteria. These five constitute the core of the origin of the CAZymes identified in the four ADs (Fig. 4 and Additional file 3), and in order to get a better understanding of which of these phyla it would be promising to look into for discovery of novel *α*-glucans-, xylans-, and cellulose-acting CAZymes.

### Cellulytic enzymes

ADs fed with primary sludge from wastewater treatment plants contain large amounts of cellulose, which stems mainly from toilet paper and constitutes about 35% of the suspended solids in the influent [56]. This is reflected in the vast number of cellulytic enzymes identified in all four ADs (Fig. 3a). The cellulose found in toilet paper is highly accessible to hydrolytic enzymes due to the extensive chemical and heat treatments during production [57, 58] and may therefore be less recalcitrant than when it originates directly from the natural source. It is therefore not surprising that the cellulytic enzymes identified in the four ADs studied here were limited almost exclusively to endoglucanases (EC 3.2.1.4) that randomly hydrolyze the *β*-1,4-linkages in amorphous regions of cellulose [59], and *β*-glucosidases (3.2.1.21) (Fig. 3a) that hydrolyze the same linkage in cellobiose and cello oligosaccharides thereby producing glucose [59]. The endoglucanases identified in the four ADs are mainly from GH5 and GH9 (Additional file 4), similar to what is seen in other anaerobic habitats [17, 42, 60]. A few endoglucanases belong to GH6, GH8, GH44, GH45, and GH51 (Additional file 4). The *β*-glucosidases are mainly from GH1 and GH3 (Additional file 4), which is also the case in other anaerobic environments [17, 42, 60], and a single gene stems from GH116 (Additional file 4). Cellulytic enzymes from bacteria are sometimes found in cellulosomes, which are enzyme complexes consisting of several cellulytic enzymes and other proteins involved in cellulose degradation [6]. However, no dockerin domains were identified in the cellulytic enzymes identified in the four ADs (Additional file 5) suggesting that the cellulases in the ADs studied operates independently.

**Fig. 5:**
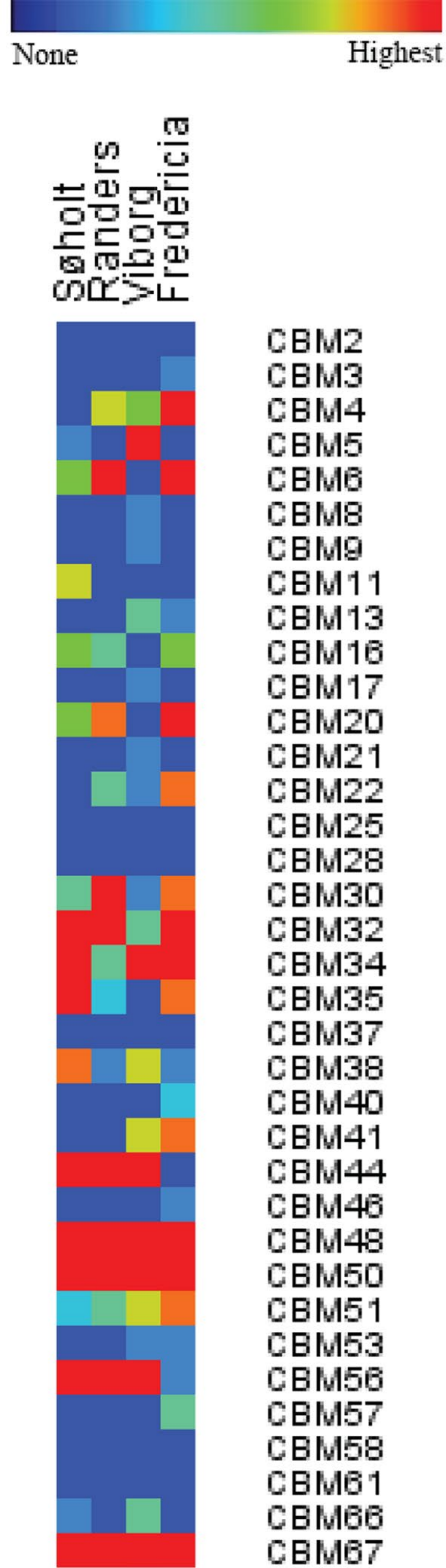
Identified carbohydrate-binding modules: heat map of all the identified carbohydrate-binding modules (CBMs) in the metagenomes from the anaerobic digesters (ADs) Søholt, Fredericia, Randers, and Viborg. The CBM families are listed on the *right side* and named according to the Carbohydrate-Active Database (http://www.cazy.org). The heat maps are calculated individually for each AD. CBM families with no identified members in any of the metagenomes are not shown

Many CAZymes secreted into the sludge by the microorganisms are trapped in the exopolysaccharide matrix synthesized by the microorganisms [61], which likely ensures that the CAZymes remain close to the cell, hence also the produced glucose, which also limits the usefulness of cellulosomes. Additionally, CBMs from CBM3, CBM4, CBM17, CBM28, CBM30, and CBM46 known to bind to non-crystalline cellulose were appended to some of the cellulytic enzymes (Fig. 5; Additional file 4).

**Fig. 6:**
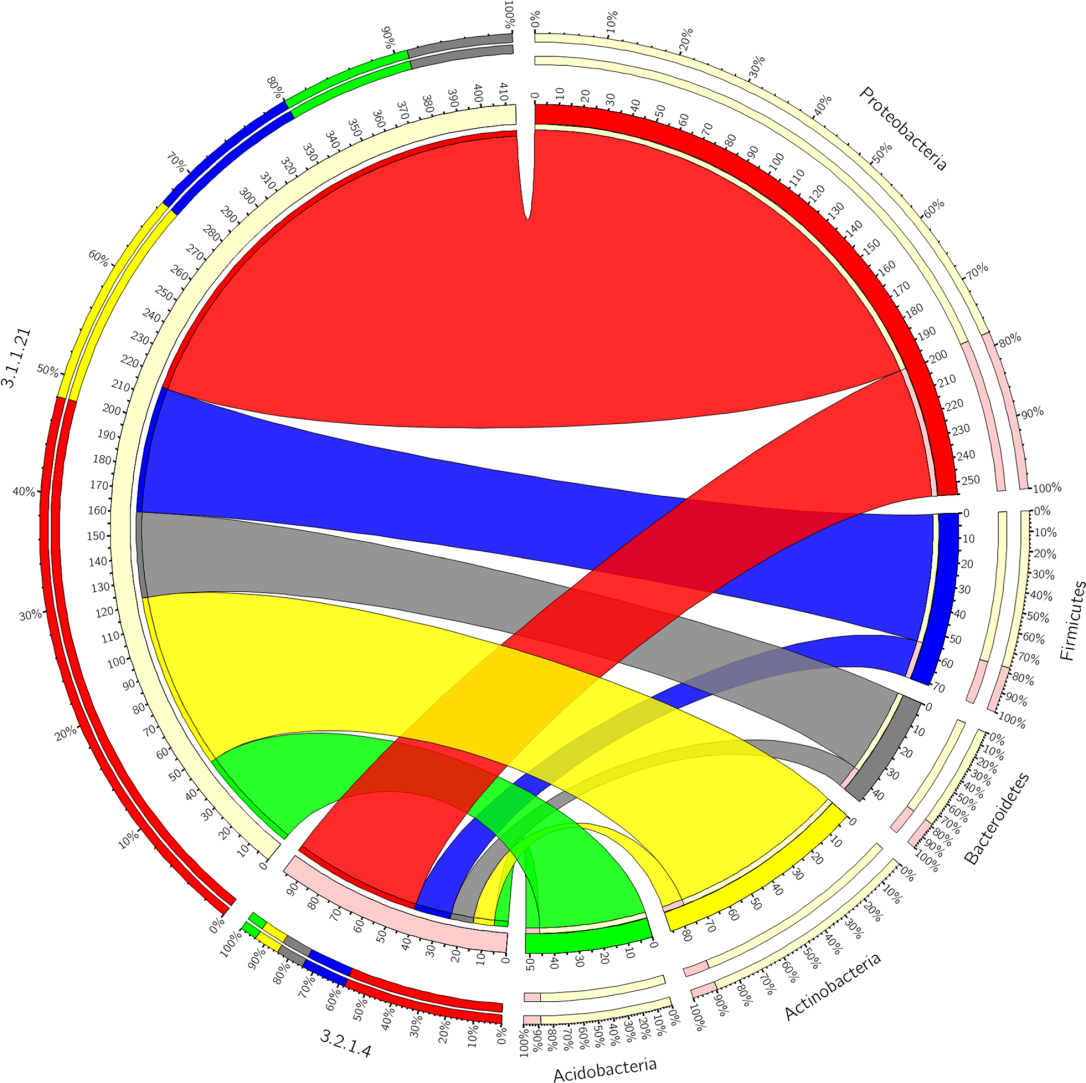
Phyla mainly responsible for cellulose degradation in the anaerobic digesters: Phylogenetic distribution of phylum level of Homology to Peptide (HotPep)-predicted cellulytic enzymes in the five phyla with highest number of HotPep-predicted cellulytic enzymes. Enzyme Commission (EC) numbers are on the *left* and the phyla are on the *right side*. The* outer ring* designates the relative abundance of genes from a given phylum (*left*) and the relative abundance of a given cellulytic enzyme (*right*); the* inner ring *designates the total number of genes encoding for a given cellulytic enzyme (*left*) and the total number of cellulytic enzyme associated with the given phylum. The *width of the bars* between a given phylum and a given cellulytic enzyme indicates their relative abundance to the other phyla mentioned

The majority of the *β*-glucosidases and endoglucanases are from Proteobacteria (Fig. 6). Interestingly, most Proteobacteria are opportunist, which means that they do not harbor genes encoding for both *β*-glucosidases and endoglucanases [62], and when searching for novel independently operating *β*-glucosidases and endoglucanases in the four metagenomes, the genes originating from Proteobacteria will be a good starting point.

### Xylanolytic enzymes

Xylans are often more complex than cellulose in the sense that the *β*-1,4-xylose backbone is often decorated with, e.g., single and/or double substituted *α*-1,2- and/or *α*-1,3-arabinofuranose that can be decorated with ferulic acid. 4-*O*-methyl-d-glucuronic acid linked *α*-1,2 to the xylose is another common substitution and so is single and/or double substituted acetyl linked *O*-2, *O*-3 and/or *O*-4 to the xylose. The position, type, and amount of substitutions vary greatly with the origin of the xylan [63–66]. Hence, many different CAZymes are needed for complete saccharification of xylans [65–68], and several of these are present in the four ADs in great numbers. Whether xylan is present or not in the sludge fed into the four ADs is unknown. However, xylan could be present in the vast amount of degrading toilet paper present in the municipal wastewater [57, 58].

Xylanolytic enzymes are present in great numbers in the four metagenomes, especially xylanases (EC 3.2.1.8) and *β*-xylosidase (EC 3.2.1.37). Both of these hydrolyze 1,4-*β*-d linkages between the xylose residues constitute the main chain of xylan [69]. The majority of the xylanases are of GH10; however, some are of GH8 (Additional file 4). The substrate specificity of GH10 xylanases is heteroxylans with a high degree of substitution, while GH8 xylanases have a more diverse substrate specificity, but with limited activity towards highly substituted xylans [70], suggesting that the microorganisms encounters both types of xylans. The less substituted xylans may also be a product of the xylanolytic accessory enzymes mentioned below, which then allows the GH8 to degrade the xylan main chain. *β*-xylosidase hydrolyzes the oligosaccharides produced by the xylanases into xylose or smaller oligosaccharides [66], and in general these are classified as GH3, GH39, GH43, and GH120 members for all the ADs (Additional file 4).

Xylanolytic accessory enzymes like *α*-l-arabinofuranosidases (EC 3.2.1.55), *α*-glucuronidases (EC 3.2.1.139), acetyl xylan esterases (AXEs) (EC 3.1.1.72), and ferulic acid esterases (FAEs) (EC 3.1.1.73), which are important for degradation of xylan from various sources [59], are all present in the four metagenomes (Fig. 3a–b).

Among the many FAEs identified in the four ADs, there are several FAEs linked to a CBM48 (Additional file 5), which are known to bind *α*-glucans like starch [71] that to the best of our knowledge does not contain ferulic acid. One could speculate that some of the bacteria in the ADs produce *α*-glucan exopolysaccharides (EPS) that are ferulated, which to the best of our knowledge have not been shown to exist.

Several xylanolytic CAZymes consisting of two or more catalytic domains are present in the four metagenomes (Additional file 5), which ensure that the functions required for degrading highly substituted xylans are in close proximity when secreted into the EPS matrix. Additionally, CBMs are in some cases appended to both these multidomain xylanolytic enzymes and individual domains (Additional file 5).

**Fig. 7:**
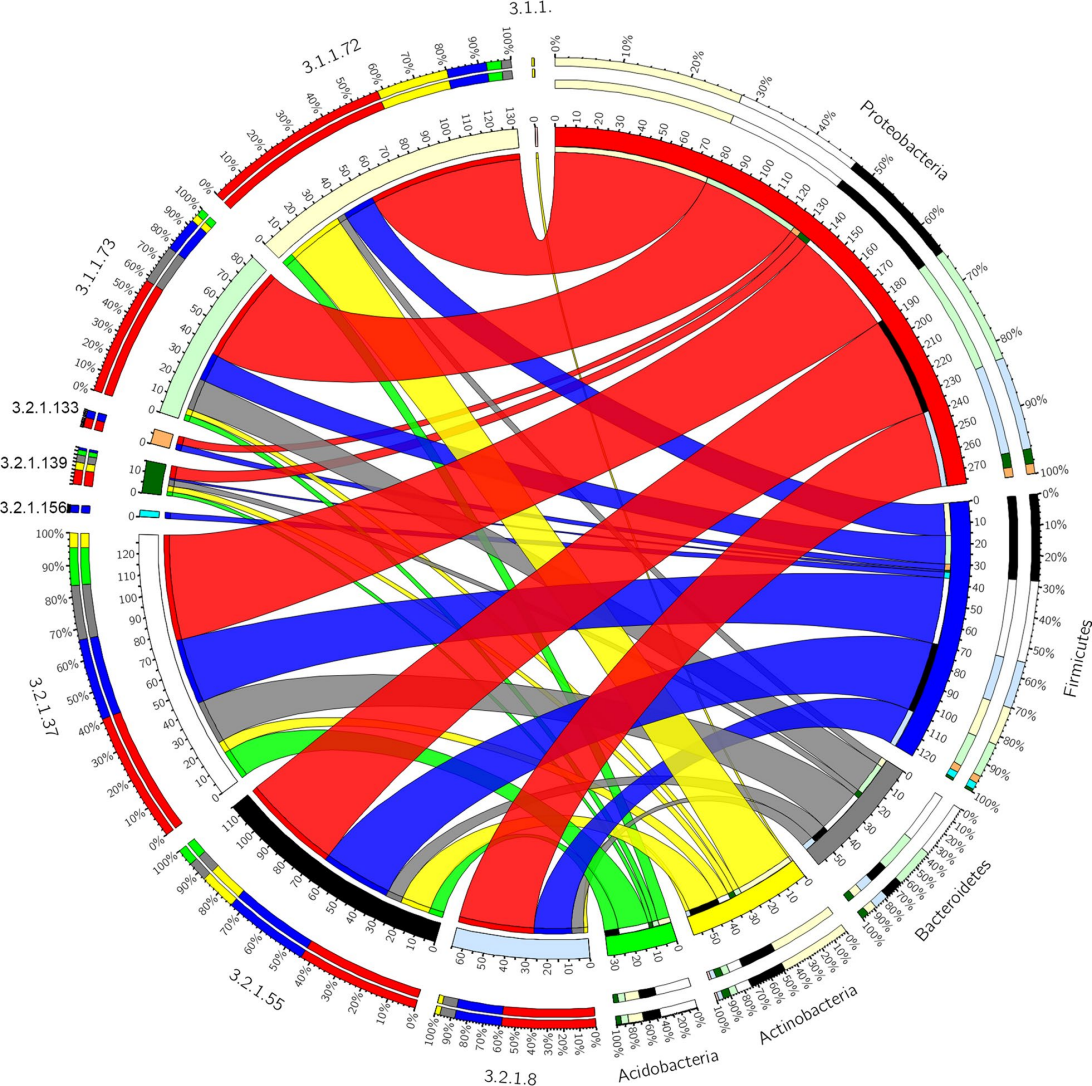
Phyla mainly responsible for xylan degradation in the anaerobic digesters: Phylogenetic distribution of phylum level of Homology to Peptide (HotPep)-predicted xylanolytic enzymes in the five phyla with highest number of HotPep-predicted xylanolytic enzymes. Enzyme Commission (EC) numbers are on the *left* and the phyla are on the *right side*. The* outer ring* designates the relative abundance of genes from a given phylum (*left*) and the relative abundance of a given xylanolytic enzyme (*right*); the* inner ring* designates the total number of genes encoding for a given xylanolytic enzyme (*left*) and the total number of xylanolytic enzyme associated with a given phylum. The *width of the bars* between a given phylum and a given xylanolytic enzyme indicates their relative abundance to the other phyla mentioned

Proteobacteria is the dominating phylum when it comes to xylanolytic CAZymes, although it accounts for a smaller relative abundance of the xylanolytic than of the cellulytic CAZymes (Fig. 7). Acidobacteria has all the required CAZymes to degrade arabinoglucuronoxylan except endo-1,4-*β*-xylanases (Fig. 7), which are essential for xylan degradation.

### Glucan active enzymes

GH13 is the most abundant GH family in the four metagenomes (Fig. 2a; Additional file 1). GH13 is a family of *α*-glucan-acting CAZymes [16] suggesting that the microbial communities encounter *α*-glucans (e.g., starch and glycogen). Glycogen-accumulating bacteria are abundant in ADs [72, 73] and GH13 CAZymes are necessary for hydrolysis of these polysaccharides when the glucose is needed for energy [16, 74]. However, this alone may not explain the large number of *α*-glucan-degrading CAZymes (Fig. 3a) and many of the genes encoding for GH13 enzymes are not of glycogen-accumulating bacteria. Further, many of the GH13 enzymes are extracellular (data not shown). Some bacteria produce *α*-glucan EPSs though [75, 76], which other bacteria could utilize as a carbon source if their *α*-glucan-acting CAZymes can hydrolyze the EPSs. Very complex EPS could potentially be present in the four ADs. Complete degradation of these would therefore require a large number of different CAZymes, which can also be found in the metagenomes from the four ADs (Fig. 3a). These EPSs may differ significantly from known polysaccharides, which may have resulted in evolution of novel functions of *α*-glucan-acting CAZymes in order to enable the bacteria to degrade the EPSs.

**Fig. 8:**
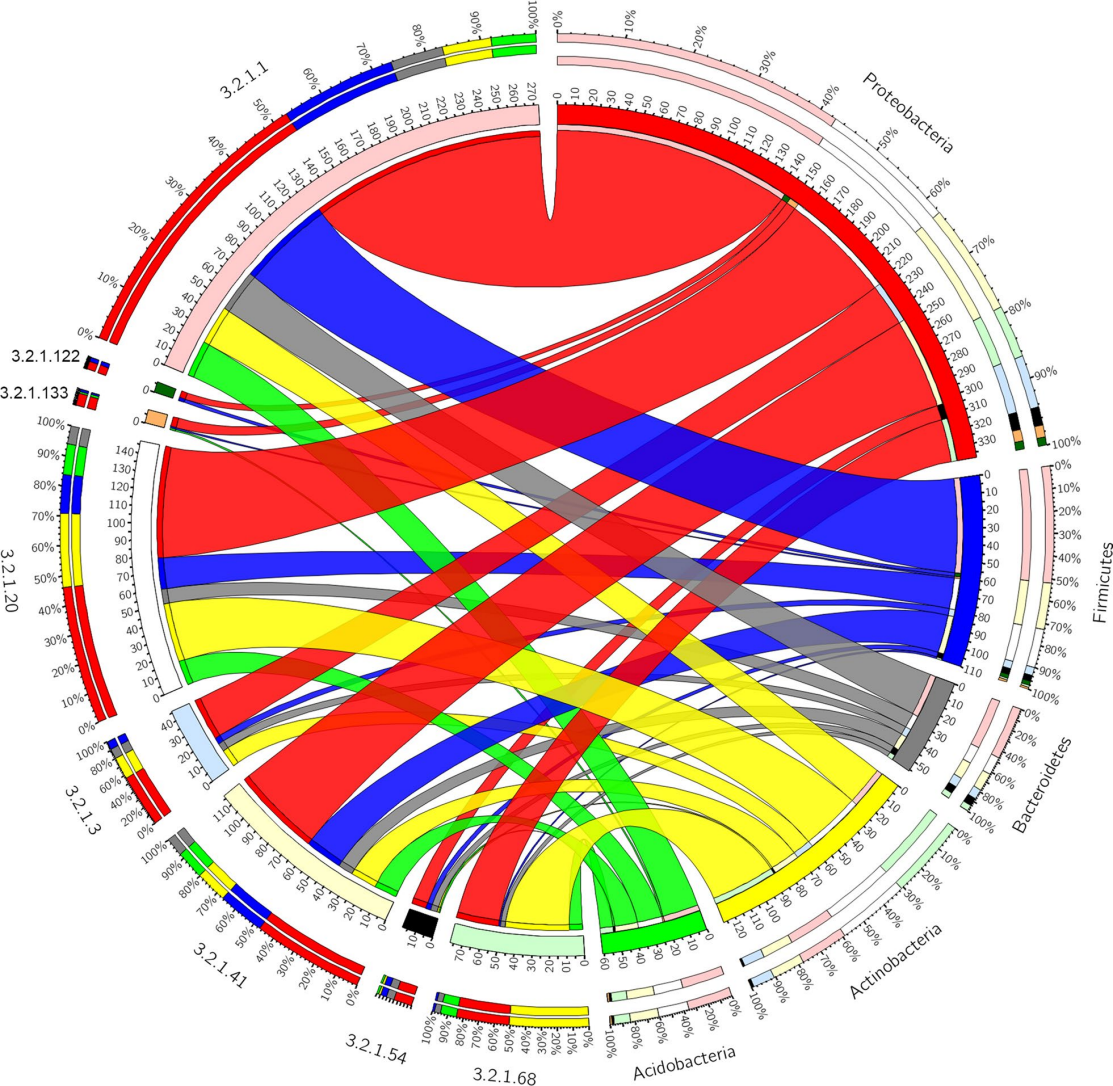
Phyla mainly responsible for α-glucan degradation in the anaerobic digesters: Phylogenetic distribution of phylum level of Homology to Peptide (HotPep)-predicted *α*-glucan-degrading enzymes in the five phyla with highest number of HotPep-predicted *α*-glucan-degrading enzymes. Enzyme Commission (EC) numbers are on the *left* and the phyla are on the *right side*. The* outer ring* designates the relative abundance of genes from a given phylum (*left*) and the relative abundance of a given *α*-glucan-degrading enzyme (*right*); the* inner ring* designates the total number of genes encoding for a given *α*-glucan-degrading enzyme (*left*) and the total number of *α*-glucan-degrading enzyme associated with a given phylum. The *width of the bars* between a given phylum and a given *α*-glucan-degrading enzyme indicates their relative abundance to the other phyla mentioned

Species of Proteobacteria and Firmicutes are represented in all the *α*-glucan-degrading CAZymes functions identified in this study (Fig. 8). Bacteroidetes, Actinobacteria, and Acidobacteria are not represented in all the *α*-glucan-degrading CAZymes functions (Fig. 8). However, it is difficult to predict in which phyla superior *α*-glucan-acting CAZymes can be found due to our lack of understanding of their presumed substrate.

## Conclusion

We identified nearly 20,000 CAZymes in the four ADs studied here and 30% of them showed 50% or lower identity to known proteins. This suggests that ADs fed with primary sludge and surplus sludge from municipal wastewater treatment plants are in fact a unique place to look for novel CAZymes. Further, we were only able to assign a function to 54% of the CAZymes by our unique bioinformatics approach, which demonstrates that there is still much to uncover about the functionality of CAZymes as HotPep only assigns a function to a PPR group if a CAZyme within the group have already been characterized. This suggests that the ADs are a good place to look for novel plant biomass degrading and modifying enzymes that can potentiate biological processes and provide basis for production of a range of added-value products from biorefineries.

## Additional files

**Additional file 1:** **Dataset S1.** Closets hit for identified CAZymes determined by BLASTP.

**Additional file 2:** Clostets hits for identified CAZymes in the ADs studied here determined by BLASTP.

**Additional file 3:** **Dataset S2.** Phylopythia output.

**Additional file 4:** **Dataset S3.** Overview of identifed specificities within the anaerobic digesters and their CAZy family number.

**Additional file 5:** **Dataset S4.** dbCan identified domains in the HotPep identifed CAZymes.
